# Tracking Microbial Diversity and Hygienic-Sanitary Status during Processing of Farmed Rainbow Trout (*Oncorhynchus mykiss*)

**DOI:** 10.3390/foods12203718

**Published:** 2023-10-10

**Authors:** Salud María Serrano Heredia, Javier Sánchez-Martín, Verónica Romero Gil, Francisco Noé Arroyo-López, Antonio Benítez-Cabello, Elena Carrasco Jiménez, Antonio Valero Díaz

**Affiliations:** 1 Department of Food Science and Technology, UIC Zoonosis y Enfermedades Emergentes (ENZOEM), CeiA3, Universidad de Córdoba, Campus Rabanales, 14014 Córdoba, Spain; t52sehes@uco.es (S.M.S.H.); t52samaj@uco.es (J.S.-M.); z32rogiv@uco.es (V.R.G.); bt2vadia@uco.es (A.V.D.); 2 Food Biotechnology Department, Instituto de la Grasa (CSIC), C\Utrera Km 1, Campus Universitario Pablo de Olavide, Building 46, 41013 Seville, Spain; fnoe@ig.csic.es (F.N.A.-L.); abenitez@ig.csic.es (A.B.-C.)

**Keywords:** spoilage microorganisms, fish farm, food contact surfaces, water, environment, viscera, flesh, aquaculture

## Abstract

Aquaculture is becoming a strategic sector for many national economies to supply the increasing demand for fish from consumers. Fish culture conditions and processing operations can lead to an increase in microbial contamination of farmed fish that may shorten the shelf-life of fish products and byproducts, and ready-to-eat fishery products. The objective of this study was to evaluate the hygienic-sanitary status of water, environment, and processing of fresh-farmed rainbow trout (*Oncorhynchus mykiss*) fillets produced in a local fish farm in Andalusia, Spain. To achieve this, a longitudinal study was carried out by collecting environmental (air and food-contact surfaces), water from fish ponds, and rainbow trout samples. Thereby, seven sampling visits were performed between February 2021 and July 2022, where foodborne pathogens and spoilage microorganisms, together with physicochemical parameters, were analysed in the collected samples. Further, microbial identification of microbiota was achieved through a culture-dependent technique using blast analysis of 16S RNA gene sequencing. The results showed that *Listeria monocytogenes* and *Salmonella* were not detected in the analysed samples. Regarding the hygienic-sanitary status of the fish farm, the slaughtering bath, the eviscerating machine and the outlet water from fish ponds presented the highest counts of coliforms, *Enterobacteriaceae*, and Aerobic Mesophilic Bacteria. *Staphylococcus aureus* and sulphite-reducing *Clostridium* were identified in the conveyor belts, fish flesh, and viscera. The 16S RNA identification confirmed the presence of viable spoilage bacteria such as *Citrobacter gillenii*, *Macrococcus caseolyticus*, *Hafnia paralvei*, *Lactococcus lactis*, *Lactococcus cremoris*, *Klebsiella*, *Escherichia coli*, *Morganella morganii*, and *Shewanella*. Three of these genera (*Citrobacter*, *Hafnia*, and *Pseudomonas*) were present in all types of samples analysed. The results evidenced potential transmission of microbial contamination from contaminated packaging belts and boxes, evisceration and filleting machines to flesh and viscera samples, thus the establishment of control measures should be implemented in fish farm facilities to extend the shelf-life of farmed fishery products.

## 1. Introduction

The increasing demand for fishery products makes aquaculture one strategic sector for the farming and commercialisation of aquaculture organisms such as fish, mollusks, crustaceans, and algae on the coast and inland, reducing pressure on wild fish populations. According to FAO, aquaculture contributes to the efficient use of natural resources, food safety, and development of the economy, with a limited and controlled impact on the environment [1]. In terms of nutritional benefits, rainbow trout is considered very heart-healthy and nutritious; it is a low-fatty oily fish (3%), with most of its fatty acids polyunsaturated, omega-3, and unsaturated, it is rich in vitamins B3, B6, B12, and D and minerals such as selenium and phosphorus [2]. 

In the European Union (EU) in 2019, aquaculture production increased to 1,141,290 t, of which 191,262 t corresponded to rainbow trout, which was the third species in production and the first in value. Moreover, Spain was the member state with the largest aquaculture production, with 308,033 t (27% of the EU) [3]. In 2021, aquaculture production increased by 19,276 t in Spain, but rainbow trout production decreased considerably with a total production of 15,357 t. Consumption per capita of aquatic products in the EU in 2019 was 24.1 kg/person, while only 6.27 kg per capita corresponded to aquaculture, 0.41 kg per person in the case of rainbow trout [1]. In Spain, 70% of the rainbow trout comes from inland production (168 inland farms), built on the banks of rivers and where the circulation by gravity of fresh water is used to renew and oxygenate the water and to avoid contamination or sediment [1], as is the case of the fish farm under study. Quality requirements of water intended to be used in fish farming factories should have a carrying capacity between 18 and 25 kg/m^3^, with a dissolved oxygen concentration between 7.5 and 12 ppm, temperature between 13 and 18 °C, pH between 6.5 and 8.5 with a minimum water height of 60–80 cm [4]. Furthermore, in the production ponds, trout should be classified by size to avoid cannibalism among them, and the water conduction system daily supervised to detect possible leaks or blockages in its passage, since the lack of water for a few minutes can cause high mortalities or predispose the fish to diseases [5]. 

Fish muscle tissue is usually free of microorganisms at the time of catching since bacteria are normally present on the skin, mucus, digestive tract, gills [6], and internal organs such as the kidney, liver, and spleen of healthy fish [7]. The composition of the natural microbiota and the presence of pathogens in fish are related to the production and feeding regimes, culture techniques, and water environmental conditions [6,8,9,10,11,12,13]. Poor general hygienic or environmental conditions may be associated with an increased risk of exposure to a pathogen [14,15] and/or reduced product shelf-life due to microbial spoilage [16]. These conditions can be evaluated by testing food hygiene indicator microorganisms such as aerobic mesophilic bacteria (AMB) as a general indicator of microbiological quality, and *Enterobacteriaceae*, coliforms, and *Escherichia coli* for the assessment of inadequate hygiene practices and enteric contamination [17,18,19,20,21]. In global terms, the microbial ecology of rainbow trout may include groups like lactic acid bacteria (LAB) with genera like *Lactococcus* [22] or *Lactobacillus*, and other genera like *Bacillus*. [23,24], isolated both from the gastrointestinal tract of salmonids [11,25] and salmon faeces [26]. They are recognised as spoilage bacterial species but can also have a bioprotective effect against fish pathogens [23]. Balcázar et al. [27] identified LAB strains such as *Carnobacterium maltaromaticum*, *Lactobacillus curvatus*, *Lactobacillus sakei*, *Lactobacillus plantarum*, *Lactococcus lactis* subsp. *cremoris*, *Lactococcus lactis* subsp. *lactis*, *and Leuconostoc mesenteroides.* Presence of pathogenic bacteria and/or spoilage microorganisms have been identified in rainbow trout, such as *Escherichia coli*, *Clostridium botulinum*, *Aeromonas* [23,28,29,30], *Macrococcus* [24], *Enterobacter* [23], *Yersinia ruckeri* [31], sulphite-reducing *Clostridium* (SRC), *Pseudomonas*, *Photobacterium*, *Shewanella*, *Vibrio*, together with some yeasts and molds [32,33,34], *Salmonella* [35] and *Listeria monocytogenes* [36] specifically in water and culture surfaces and in gills, skin and viscera of rainbow trout.

To avoid the spread of microbial contamination during the catching, transformation, storage, and distribution of fish farm rainbow trout, good hygienic practices must be followed together with storage at refrigerated conditions (<4 °C). This will prevent the fish meat from rapidly decomposing due to microbial activity and contribute to extending the shelf-life of trout fillets [4,28]. Since little information is available in the literature on the hygienic-sanitary status of fresh rainbow trout, it would be relevant to shed light on the distribution of bacterial groups and species and contamination routes during product transformation in fish farm factories. This study aimed to carry out a systematic environmental and product sampling to evaluate the microbial status of farmed rainbow trout (*Oncorhynchus mykiss*) produced in a freshwater continental fish farm in Andalusia (Spain). Further, a molecular identification of the main pathogenic and/or spoilage species was achieved to study the origin and potential routes of transmission/cross-contamination along the fish production chain. Finally, food safety implications in the finished products have been discussed. The results obtained will be useful for risk managers in order to establish adequate measures to mitigate microbial risks during the production of fresh-farmed rainbow trout and products thereof. 

## 2. Materials and Methods

### 2.1. Sampling and Experimental Design

**Figure 1:**
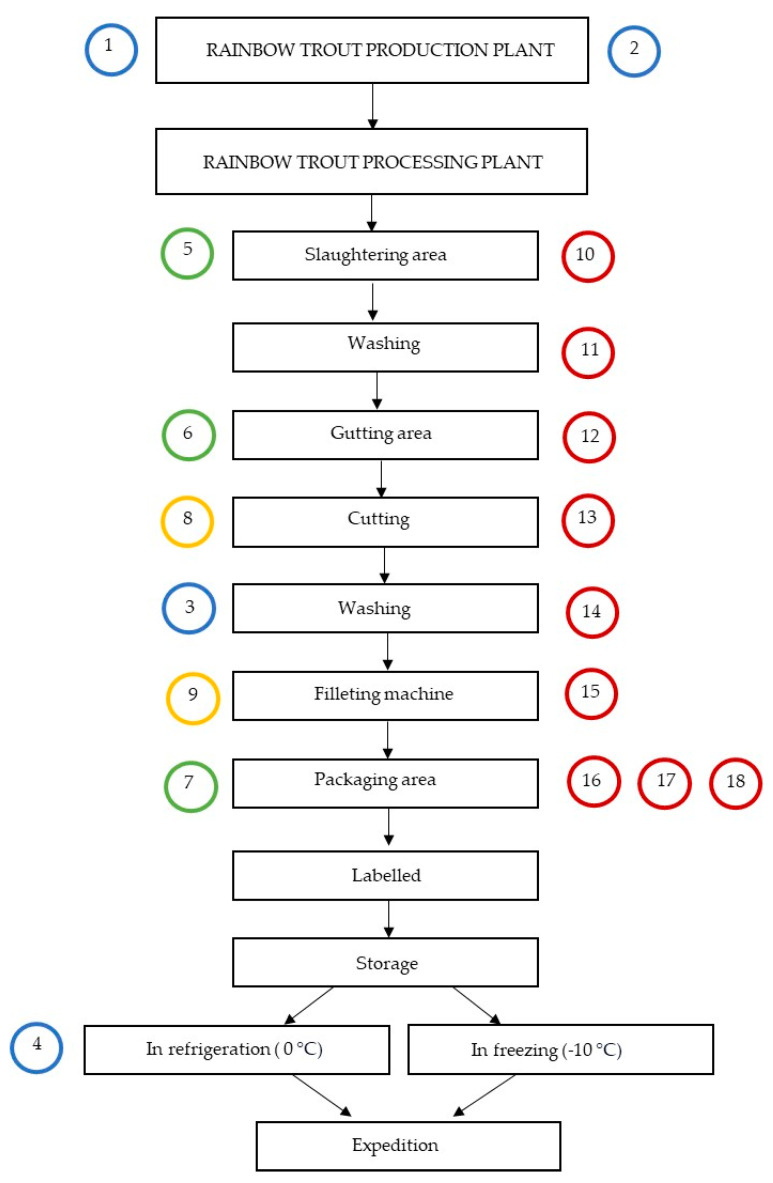
Flow diagram of the main industrial operations of fish-farmed rainbow trout. Collected samples of water, environmental air, surfaces, flesh, and viscera of rainbow trout are illustrated as follows. Water samples (blue circles)—1: Input water, 2: Output water, 3: Processing water, 4: Ice samples. Environmental samples (green circles)—5: Slaughtering area, 6: Gutting area, 7: Packaging area. Rainbow trout samples (yellow circles)—8: Rainbow trout viscera sample, 9: Rainbow trout flesh sample. Food-contact surfaces samples (red circles)—10: Slaughtering bath, 11: Washing belt, 12: Gutting machine, 13: Knife, 14: Worker hands, 15: Filleting machine, 16: Packaging belt, 17: Packaging box, 18: Packaged fillets belt.

A longitudinal study was carried out in a freshwater continental fish farm factory located in southern Spain. A total of 154 samples obtained from seven visits, were collected during a 20-month period (April, October, November 2021, January, April, July, and November 2022), classified into four different categories: water (n = 28), environmental air samples (n = 42), food-contact surfaces (n = 63), flesh (n = 21) and viscera (n = 21) from processed rainbow trout fillets coming from multiple rearing groups. The seasonal variations between samplings have been considered a source of microbial variability since water quality parameters, as well as the microbiota composition of the rainbow trout, may be affected by temperature and sunlight changes. For the purposes of this study, “seasonality” refers to the period during which samples were collected. The main industrial operations and the specific sampling points are indicated in the flow diagram described in Figure 1.

At each sampling, water samples were taken at the input (n = 1) and the output locations (n = 1) of the trout farming ponds. In addition, the processing water sample (n = 1) and ice sample (n = 1) were taken from the transformation plant, as shown in Figure 1. For water sampling, sterile glass bottles with a volume of 1 L were used, while a sterile duchess was used for ice.

Additionally, environmental air samples (n = 6) were taken from three different areas (slaughtering area, gutting area, and packaging area), with the aid of an air sampler (SAS Super 100 Air Sampler, Bioscience 2 International^®^, Rockville, MD, USA), as indicated in Figure 1. was used. Samples were taken sequentially from the same sites throughout the sampling period and collected by the same person to control reproducibility and repeatability. A total air volume of 200 L was collected at each site for enumeration of total AMB and yeasts and molds, using plate count agar (PCA, Oxoid, UK) and rose-bengal chloramphenicol agar (RBCA, Oxoid, UK) plates.

Flesh (n = 3) and viscera (n = 3) samples of rainbow trout were taken at each sampling by using sample collection bags and sterile duchesses, respectively.

Surface samples (n = 9) were taken at the rainbow trout processing and transformation plant at each sampling, selecting rough and hard-to-access surfaces (where cleaning and disinfection procedures are most likely to be ineffective), equipment, utensils, and the hands of a worker (Figure 1). Surface samples (n = 9) were taken using a sterile sampling swab previously moistened with sterile 0.1% peptone water (Oxoid, UK). The area covered by the swab was approximately 100 cm^2^. Swabs were placed in 4 mL peptone-water tubes and stored at 4 °C prior to microbial analysis.

All samples were transported at refrigerated temperatures in thermal boxes with ice (<4 °C) to the laboratory and were analysed within 24 h from the reception.

### 2.2. Physicochemical Analysis

Physicochemical parameters measured in the water samples were pH, conductivity, salinity, and Total Dissolved Solids (TDS) using a multiparameter water quality meter (PCI Instruments, Southport, United Kingdom). Free chlorine was measured by using a chlorine photometer (Hanna Instruments^®^, Singapore). In product samples of rainbow trout (flesh and viscera), pH was measured by using a calibrated pHmeter equipment with an insertion electrode (Edge multiparameter, Hanna Instruments, Madrid, Spain). Water activity (a_w_) of the flesh samples was measured using a calibrated Aqualab equipment (AQUALAB Serie 4, MeterFood^®^, Pullman, WA, USA).

### 2.3. Microbial Analysis

The standards and methods for microbial analysis followed are shown in Table 1. 

Water samples were microbiologically analysed for the determination of total AMB, LAB, total coliforms, *Enterobacteriaceae*, SRC, and *L. monocytogenes*. Results were expressed in CFU/mL.

Environmental air samples were microbiologically analysed for AMB, and yeasts and molds (YM) [47]. Results were adjusted from probable counts to colony-forming units per cubic meter (CFU/m^3^) of sampled air, following the air sampler instructions.

Surface samples were microbiologically analysed for AMB, *Enterobacteriaceae*, total coliforms, coagulase-positive *Staphylococcus*, and *L. monocytogenes*. Samples were cultured using a spiral plater (IUL Instruments, Spain) and incubated at the appropriate temperatures. Subsequently, counts were carried out with the help of an automatic colony counter (Flash & Go, IUL; Barcelona, Spain), expressing the results in CFU/cm^2^. Prior to analysis, all tubes containing sampled swabs were shaken to facilitate the release of microorganisms into the broth culture. For *L. monocytogenes* detection, the two-step enrichment method based on ISO 11290–1 [46] was carried out. Environmental swabs were enriched in 4 mL of half-Fraser broth (HF) for each tube. After 24 h incubation, 100 mL of HF enrichment was spread onto plates and likewise 1:10 in full-strength Fraser broth (FF) [48]. The final concentration of microorganisms was expressed as CFU/cm^2^.

Rainbow trout flesh and viscera samples were microbiologically analysed for AMB, YM [47], *Enterobacteriaceae*, *Enterococcaceae*, coagulase-positive *Staphylococcus*, LAB, psychrotrophic bacteria, *Salmonella*, SRC, and *L. monocytogenes*. For the analysis, flesh, and viscera samples were cut with a sterile scalpel under aseptic conditions. Next, 10 g of muscle and viscera were weighed in sterile stomacher bags (Seward^®^, Easting Close Worthing West Sussex, UK), 1:10 diluted with 90 mL of diluent using a gravimetric dilutor system (IUL Instruments^®^, Barcelona, Spain), and homogenised in a blender (IUL Instruments, Spain) for 60 s at 1500 rpm. The diluents used were peptone water (PW, Oxoid, UK) and *Listeria* fraser broth (Oxoid) (the latter for the analysis of *L. monocytogenes*). Samples were cultured using a spiral plater (IUL Instruments, Barcelona) and incubated at the appropriate temperatures (Table 1). After incubation, colonies grown on plates were enumerated using an automatic counter (Flash & Go, IUL; Barcelona, Spain) to finally express the results in CFU/g of the sample.

### 2.4. Isolation and Molecular Identification

Based on the results obtained for the microbial groups analysed, the different colonies with typical morphology of *Enterobacteriaceae*, coliforms, *Enterococcus*, LAB, SRC, coagulase-positive *Staphylococcus*, *Salmonella*, *L. monocytogenes*, and *Aeromonas* were randomly selected from water, food-contact surfaces, and rainbow trout samples (flesh and viscera). All colonies obtained with the characteristic morphology of each microorganism analysed were selected. Then, all colonies were purified on tryptone soy agar culture medium (TSA, Oxoid, UK) to proceed with their molecular identification. 

The procedure for the molecular identification of the samples from the purified isolates included (i) the extraction of bacterial chromosomal DNA using a specific kit (InstaGene Matrix, Bio-Rad), and (ii) PCR amplification of the 16S RNA ribosomal gene using the My Taq DNA polymerase enzyme (Bioline) following the manufacturer’s recommendations. In brief, a reaction mixture was prepared with 5 µL of 5X Buffer, 1 µL of oligonucleotide 27F 5′AGAGTTTGATCMTGGCTCAG3′(10 mM), 1 µL of oligonucleotide 1492R 5′CGGTTACCTTGTTACGACTT3′ (10mM), 10 µL of template DNA, 7.8 µL of molecular water and 0.2 µL of the enzyme. DNA amplification was performed in a Mastercycler Pro S vapo.protect (Eppendorf) programmed as follows: 4 min at 94 °C; 25 cycles of 2 min at 94 °C, 2 min at 55 °C and 2 min at 72 °C; and 7 min at 72 °C. The amplification products were analysed by electrophoresis in 1% agarose gel, stained with ethidium bromide (20 min), and visualised under ultraviolet light. Subsequently, the PCR product was purified using the purification kit (NucleoSpin PCR clean-up, Macheryy-Nagel). Finally, Sanger sequencing was carried out with STAB VIDA (Caparica, Portugal) and BLAST analysis in the NCBI database to determine the most probable identities of the strains. Only sequence similarities above 97% were considered significant for bacterial identification at the species level.

### 2.5. Statistical Analysis

R Studio software (version 2022.07.2) was used for data analysis. Graphs were built using *ggstatsplot* and *ggplot* packages. To compare the microbial contamination levels among the different processing areas and seasons, a descriptive analysis (e.g., median, mean, standard deviation), ANOVA analysis, and Tukey’s Honest Significant Difference [HSD] test of the obtained microbiological data and physicochemical parameters were carried out. In addition, Pearson correlation coefficients (*r*) were calculated to establish potential relationships between environmental air, water, food contact surfaces (FCS), and product samples (*p* < 0.05).

## 3. Results and Discussion 

### 3.1. Physicochemical Assessment

The results of the physicochemical analysis of the water samples are presented in Table 2. For the pH parameter, processing water and ice had a value between 6.5 and 9.5, in accordance with the Spanish Royal Decree 3/2023, of 10 January, which establishes the technical-sanitary criteria for the quality of drinking water, its control, and supply [49]. In addition, the results obtained for the input and output water samples presented a pH within the range of 6.5–7.42, the same values obtained by other authors in farmed water of underground origin [50].

The pH results of the viscera samples analysed were 6.59 ± 0.30, while those of the flesh were 6.43 ± 0.19 and with an a_w_ value of 0.982 ± 0.001, more than 0.05 units higher than that found for flesh by Zapata et al. [51] (5.84), although very similar to the results of Díaz-Villanueva et al. [52], with a pH value of 6.5 in rainbow trout flesh after slaughtering, and the results by del Torre et. al. [53] with a pH value for flesh of 6.45 ± 0.16. 

### 3.2. Microbiological Assessment 

#### 3.2.1. Water Samples

Results of the microbiological analysis of input and output water from the fish farm ponds and processing water are shown in Table S1 (Supplementary Material). Output water presented the highest microbial counts, finding significant differences (*p* < 0.05) with input and processing water in all the microbial groups analysed. Specifically, an increase of 1.27, 1.44, 1.51, and 1.45 log CFU/mL for AMB, *Enterobacteriaceae*, LAB, and total coliforms was found in output water with respect to input water. This result can be expected since output water usually carries faeces and food from the rainbow trout farm ponds. However, no significant differences in numbers were observed between input and processing water (*p* > 0.05). 

**Figure 2:**
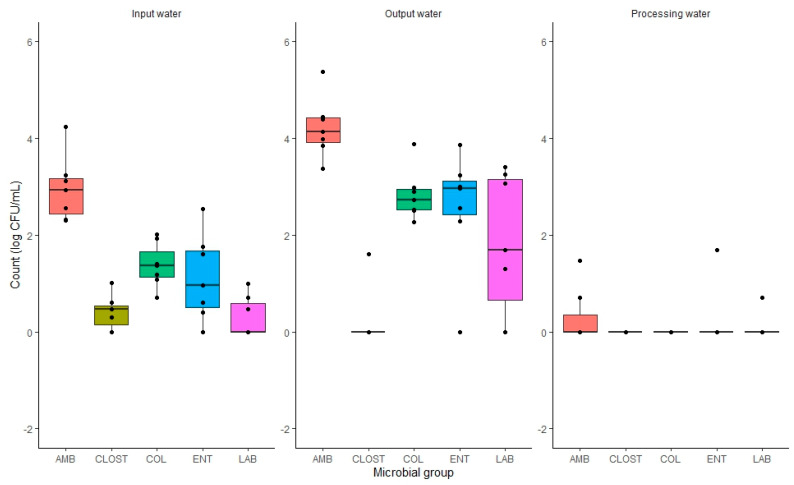
Comparison of counts (log CFU/mL) of the microbial group’s aerobic mesophilic bacteria (AMB), sulphite-reducing *Clostridium* (SRC), total coliforms (COL), *Enterobacteriaceae* (ENT) and Lactic Acid Bacteria (LAB) studied in the input, output, and processing water samples. The detection limit has been set to 1 CFU/mL (0 log CFU/mL).

Processing water presented the lowest counts for all analysed microbial groups as shown in Figure 2. This can be attributed to the microbial load of the rainbow trout, which was diluted by the washing process after slaughtering. Regarding the seasonal variations effect on the microbial loads (Table S2, Supplementary Material), there were no significant differences (*p* > 0.05) for any microorganism studied. According to the study of Grigoryan et al. [50], the bacteriological status of the water is directly related to the bacterial microbiota of the rainbow trout, constituting a source of contamination. Furthermore, the presence of coliforms in the fresh rainbow trout could indicate potential environmental contamination [54]. Finally, *L. monocytogenes* was not detected in any of the water samples analysed (limit of detection = 1 CFU/100 mL).

##### Bacterial Genus Presence in Water Samples

**Figure 3:**
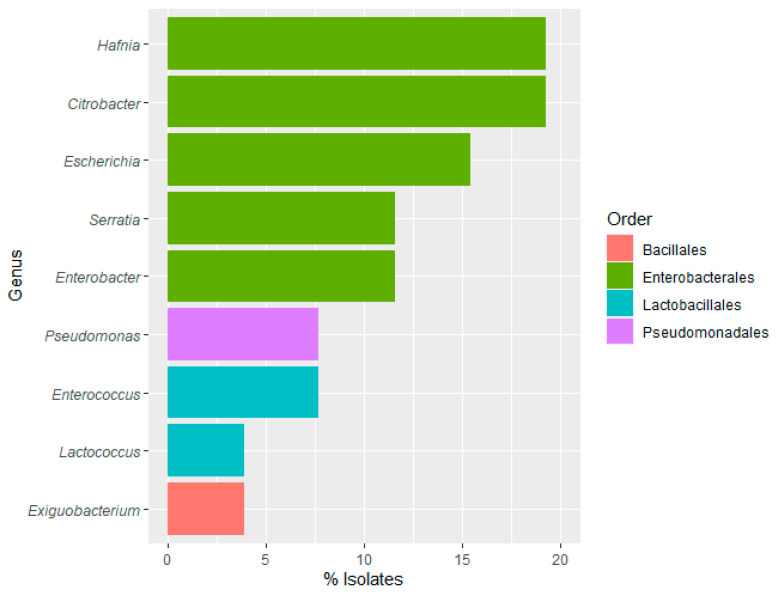
Frequency (%) of bacterial genus and orders identified in water samples.

The water samples subjected to molecular identification corresponded to both input and output water, where the highest microbial counts were obtained. Isolation was carried out through the random selection and purification of 25–50 grown colonies of AMB, *Enterobacteriaceae*, total coliforms, LAB, and sulfite-reducing *Clostridium*-specific selective growth media. Colonies showing no growth and/or unpurified cultures were discarded for further identification. Out of the 26 isolates obtained, 76.92% of the identified species belonged to the order Enterobacterales, which were present in both input and output water samples. Species of the order Lactobacillales (11.54%) were detected in the output water, Pseudomonadales (7.69%) in both input and output water, and Bacillales (3.85%) exclusively in the output water (Figure 3). 

Among the isolates of the Enterobacterales order, both *Citrobacter* and *Hafnia* were identified at a rate of 19.23% each. Notably, *Citrobacter gillenii* and *Hafnia paralvei* were the most frequently isolated species. The presence of *Citrobacter* has been associated with disease symptoms in farmed fish species, particularly in rainbow trout but also in other freshwater species [55,56]. They can be classified as faecal indicators, which suggests that their presence in water samples could be indicative of potential contamination [57]. Regarding *Hafnia*, there have been limited reports of detecting this genus in water samples [50]. However, it has been isolated from the intestinal microbiota of rainbow trout and is associated with various pathologies in farmed fish species [58]. Conversely, other studies have found that certain species of *Hafnia* are capable of producing bacteriocins, which inhibit the growth of pathogenic bacteria [59]. 

On the other hand, *Pseudomonas aeruginosa* has been isolated from the water samples in this study. *P. aeruginosa* is part of a large group of free-living bacteria that are ubiquitous in the environment and has been widely recognised as a potential pathogen for humans [60]. Prevalence cases have been reported where *P. aeruginosa* has been isolated in foods of animal origin, including fish products, and in vegetable foods and fruits [61,62,63,64]. Its detection in natural water environments, such as lakes, rivers, and ponds, appears to be primarily due to human presence and domestic activities [65,66,67]. Monitoring the microbial quality of water is essential to control the presence and spread of pathogens transmitted through contaminated water use in aquaculture farms. Additionally, the responsible use of antibiotics in fish farms and the surveillance of antibiotic susceptibility patterns are essential to prevent the rapid emergence of resistant strains of *P. aeruginosa*, which could pose a public health threat [50,68]. 

Lastly, *Lactococcus garvieae* was detected in 3.85% of the water samples, being found in the output water. This is an emerging pathogen in aquaculture, causing lactococcosis, a disease that results in significant economic losses. Moreover, it could be considered a potential foodborne pathogen for humans [69]. This species, as lactic acid bacteria, is a component of native microbiota in raw milk and derived products [70], and also, it is associated with the fermentation of fish products [71], although the disease is linked with the consumption of contaminated raw fish [72,73]. 

#### 3.2.2. Air Samples

**Figure 4:**
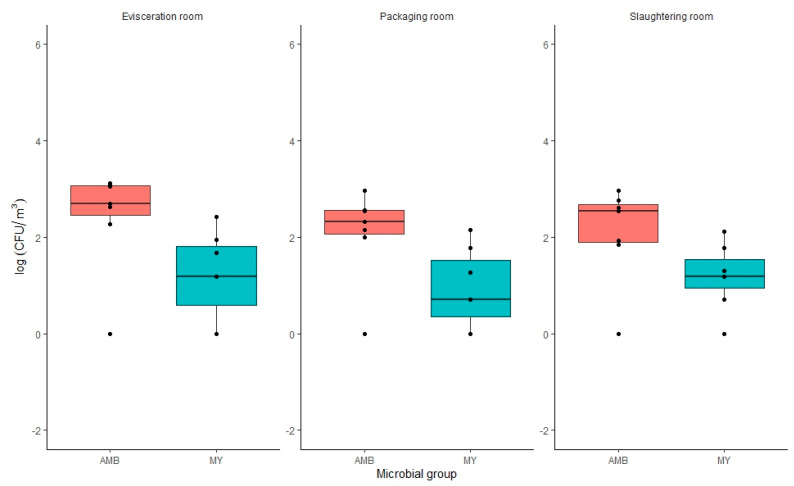
Comparison of log CFU/m^3^ counts for aerobic mesophilic bacteria (AMB) and molds and yeasts (MY) studied in the gutting, packaging, and slaughtering area. The detection limit was 1 CFU/m^3^ (0 log CFU/m^3^).

Results obtained for microbial air quality are shown in Table S3 (Supplementary Material). It can be seen that the air sampled in the gutting area presented a higher load of AMB compared to the slaughtering and packaging area, with a difference of 0.33 CFU/m^3^ (Figure 4). This could be associated with the evisceration area, where the viscera and intestines of the rainbow trout are removed, leading to an increase in the spread of microorganisms to the flesh of the fish. For molds and yeasts, the gutting area was the one with the highest counts observed, although no significant differences (*p* > 0.05) were observed with the slaughtering room (Figure 4). The packaging area presented slightly lower mean microbial counts in comparison to slaughtering or evisceration areas (Table S3, Supplementary Material).

Regarding seasonal variations, there were no significant differences (*p* > 0.05) for AMB between the seasons of spring and summer (Table S4, Supplementary Material). Nevertheless, the greatest difference (*p* < 0.05) was shown between spring and winter, with a difference of 1.52 UFC/m^3^. For molds and yeasts, there were no significant differences (*p* > 0.05) between autumn and spring. However, between summer and winter, there were significant differences (*p* < 0.05), presenting the summer period with the highest microbial counts (1.64 UFC/m^3^). For both AMB and molds and yeasts, the effect of temperature increase during summer is reflected in higher microbial loads.

To study associations between variables, and thus, potential contamination routes, Pearson correlation coefficients (*r*) were calculated between the different sampled areas. Significant correlations were found (*p* < 0.05) between the slaughtering area and the gutting area, the slaughtering area and the packaging area, and between the gutting area and the packaging area for AMB (*r* = 0.972, *r* = 0.987 and *r* = 0.937, respectively) and molds and yeasts (*r* = 0.757, *r* = 0.838 and *r* = 0.939, respectively).

These results showed that environmental air contributed to the transfer of microbial contamination in the rainbow trout processing area. For this reason, air control should be considered in the Hazard Analysis and Critical Control Points (HACCP) systems of food processing plants [74]. 

#### 3.2.3. Food-Contact Surfaces (FCS) Samples

The FCS sampling locations shown in Figure 1 were selected based on the risk of being potential sources of contamination during rainbow trout processing.

**Figure 5:**
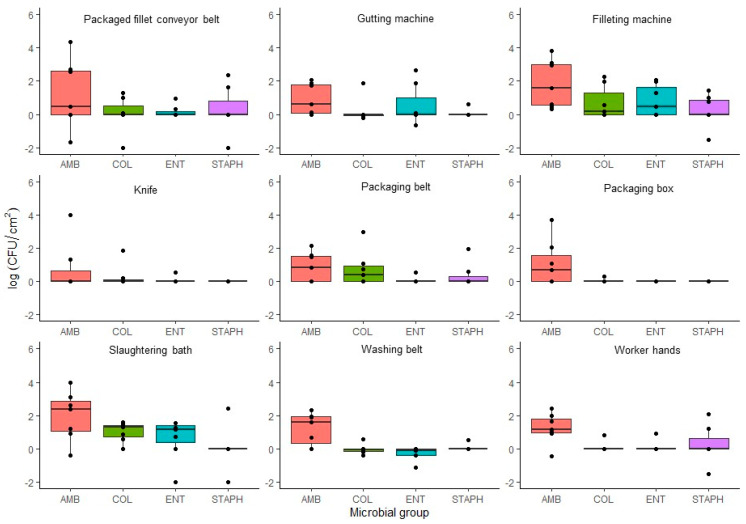
Comparison of counts (log CFU/cm^2^) for the microbial groups: aerobic mesophilic bacteria (AMB), coliforms (COL), *Enterobacteriaceae* (ENT), and coagulase-positive *Staphylococcus* (STAPH), studied in the food-contact surfaces studied. The detection limit has been set to 1 CFU/100 cm^2^ (−2 log CFU/mL).

Results of the microbiological analysis of the FCS are shown in Table S5 (Supplementary Material). The FCS that presented the highest levels of contamination for the case of AMB, coliforms, and *Enterobacteriaceae* were the slaughtering bath and the evisceration machine, with levels of 1.89 ± 0.09 CFU/cm^2^, 0.86 ± 0.21 CFU/cm^2,^ and 0.72 ± 0.16 CFU/cm^2^, respectively). However, in the case of coagulase-positive *Staphylococcus*, the most contaminated FCS were the evisceration machine, worker hands, packaging belt, and packaged fillet belt (0.30 ± 0.07 CFU/cm^2^) as shown in Figure 5.

The high AMB counts in the slaughtering bath could be due to the high microbial load of the output water as well as the microorganisms present on the rainbow trout skin. According to Figure 5 and Table S5 (Supplementary Material), the gutting machine was the FCS that presented the highest counts of *Enterobacteriaceae* and coagulase-positive *Staphylococcus*, possibly explained by the intestinal contents and viscera, with regular presence of *Enterobacteriaceae*. However, the presence of *S. aureus* may be also due to contamination through handlers. In this respect, positive results were obtained from samples taken from a worker’s hands (Table S5, Supplementary Material) just after being washed and disinfected. In addition, coagulase-positive *Staphylococcus* counts increased slightly throughout the processing chain, being higher in the FCS of the final stages such as the packaging belt and the packaged fillet belt. On the contrary, knives were the least contaminated surface by AMB despite samples having been collected immediately after cleaning and disinfection. 

The effect of seasonal variations on microbial counts in FCS is represented in Table S6 (Supplementary Material). It can be seen that, for AMB, total coliforms, and *Enterobacteriaceae*, the highest counts were obtained in the visits of autumn, while for coagulase-positive *Staphylococcus*, the highest counts occurred in summer. However, there were no significant differences (*p* > 0.05) between seasons. The correlations found between the different FCS analysed are shown in Table 3. In general, the microbiological analysis of FCS in the fish farm revealed that the slaughtering bath and the evisceration machine were the FCS that presented higher counts of AMB, coliforms, and *Enterobacteriaceae*, although at relatively low counts. This could be an indicator that cleaning and disinfection procedures are carried out correctly in the rainbow trout processing plant. Regarding the correlations found between the FCS analysed, the highest correlations for AMB were found in packaging belts and packaging boxes with evisceration and filleting machines, respectively. Contamination of *Enterobacteriaceae* and total coliforms found in knives were significantly correlated with high counts in packaging belts and filleting machines. Finally, in the case of coagulase-positive *Staphylococcus*, slaughtering, and evisceration were the most critical areas for the transmission of this contamination. 

According to the results obtained, although the hygienic-sanitary conditions of the analysed FCS were adequate, it is necessary to optimize the cleaning and disinfection procedures to reduce the risk of FCS contamination in aquaculture fishery processing plants.

##### Bacterial Genus Presence in Food-Contact Surfaces

**Figure 6:**
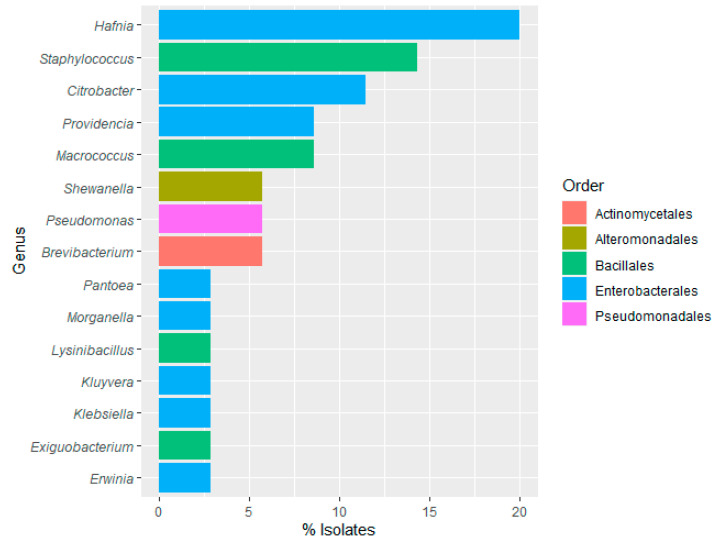
Frequency (%) of bacterial genus and orders identified in surface samples.

Regarding samples from food-contact surfaces, a total of 25–50 grown colonies were randomly selected from AMB, Enterobacteriaceae, total coliforms, and CPS-specific selective growth media. Most of the isolates corresponded to the slaughtering bath, evisceration machine, and filleting machine, where the highest microbial counts were obtained. Out of the 35 isolates identified, 54.29% belonged to the order Enterobacterales, with *Hafnia* and *Citrobacter*, being the most frequent species, (20.00% and 11.43%, respectively) (Figure 6), as previously shown with the identifications obtained in the water samples. This result highlights a potential contamination route from water to the processing line.

Subsequently, the order Bacillales represented 28.57% of the isolates, with *Staphylococcus* being the most representative genus (14.29%). Less frequently, strains of *Brevibacterium*, *Pseudomonas*, and *Shewanella* were identified (5.71% each). *Enterobacteriaceae* were present in all sampled surfaces except on the workers’ hands, where *Staphylococcus warneri* was identified. *S. aureus* were found in the evisceration machine, packaging belt, and packaged fillets belt. *Pseudomonas* was only isolated in the evisceration machine. The presence of *Staphylococcus* in food occurs frequently due to inappropriate manipulation of food by carriers of this microorganism [75]. *S. aureus*, which has been isolated from abiotic surfaces in this study, should be considered for its biofilm-forming capability on surfaces in the food industry, together with its known antimicrobial resistance properties [76]. Clearly, this factor is a serious problem in the food industry due to its impact on human health [77]. 

### 3.3. Rainbow Trout Samples

The microbiological results obtained from the analysis of flesh and viscera samples of rainbow trout are presented in Table 4. Overall, there were no significant differences between microbial counts found in viscera and flesh samples (*p* > 0.05). Further, neither *Listeria* nor *Salmonella* were detected in the analysed flesh and viscera samples. 

For the spoilage bacteria, microbial counts indicated that good aquaculture practices were followed, as most of them fell within acceptable ranges. Other studies conducted on rainbow trout have shown higher counts for the total microbiota in the intestines of adult animals, ranging between 5 and 7 log CFU/g [24], and in other fish species such as seabass, seabream, or farmed mullet (6.29, 6.14, and 6.89 log CFU/g, respectively) [78] than the average counts found in our study for flesh (3.22 log CFU/g) and viscera (3.97 log CFU/g). Psychrotrophic bacteria counts were below the established limit of 6 log CFU/g for refrigerated fishery products [79,80]. Previous works reported higher average levels (5.81–6.15 log CFU/g) in flesh and viscera from other fish species such as seabass and seabream (Boulares, 2011) [78]. LAB, generally considered favorable due to their ability to antagonize bacterial pathogens, are frequently identified as part of the gut microbiota in fish [81]. Average LAB counts in viscera (2.98 log CFU/g) and in flesh samples (2.70 log CFU/g) were below those found by Araújo (2015) [23], who obtained values above 3 log CFU/g in the intestines of adult trouts. In the study carried out by Hagi et al. [82] the composition and seasonality of intestinal LAB in freshwater cultured fish were analysed; LAB numbers varied depending on water temperature, ranging from approximately 6 log CFU/g in summer to 5 log CFU/g in winter, concluding that temperature fluctuations are likely to result in shifts in the predominant LAB strains [83]. 

Counts of *Staphylococcus* were below 0.5 log CFU/g in the rainbow trout samples. *Clostridium* counts were below 1 log CFU/g. While not common, detection and isolation of *Clostridium* have been reported in farmed fish species [84].

The genus *Aeromonas* has been implicated in food poisoning and is ubiquitous, being found in soil, water, animals, and humans. This is a psychrotrophic species that is able to grow at refrigeration temperatures and has been isolated from wild rainbow trout [30]. In our study, counts of Aeromonadaceae in the flesh and viscera samples have been relatively high (nearly 4.5–5.5 log CFU/g), consistent with the results of Naviner et al. [85]. In that study, the seasonal variability effect on the intestinal microbiota of rainbow trout was examined, with particular attention to *Aeromonas* as a potential indicator of antimicrobial resistance (>5 log CFU/g). To mitigate economic losses, preventive measures are currently being implemented, such as the use of vaccines based on outer membrane proteins of *Aeromonas* species isolated from rainbow trout [86].

#### Bacterial Genus Presence in Rainbow Trout Samples

**Figure 7:**
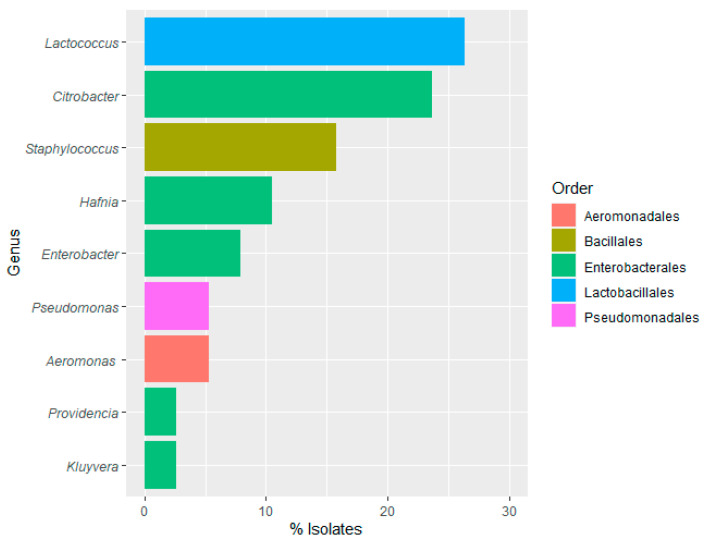
Frequency (%) of bacterial genus and orders identified in the product samples.

To proceed with the molecular identification of rainbow trout samples, 50–70 grown colonies were randomly isolated from AMB, psychrotrophic bacteria, moulds and yeasts, LAB, *Enterobacteriaceae*, *Enterococcaceae*, CPS, sulfite-reducing *Clostridium* and *Aeromonadaceae* specific selective growth media. A total of 37 isolates (20 from flesh and 17 from viscera) were selected for molecular identification. Most of them belonged to the order Enterobacterales (45.95%) (Figure 7), as previously described for water and food-contact surface samples. Within this order, *Citrobacter* was mostly identified (21.62% of the isolates). 

Identification results confirmed the presence of LAB species in the rainbow trout microbiota. The genus *Lactococcus* (27.03%) was only identified, belonging to the order Lactobacillales. Iorizzo et al. [87] studied the presence of LAB in the intestinal tract of wild Mediterranean trout, including *Lactococcus lactis*, which was also isolated in this study in the viscera. LAB plays an important role due to the production of antimicrobial substances, improvement of disease resistance, greater tolerance to oxidative stress, stimulation of the immune response, and increased nutrient availability. In contrast, *Lactococcus garvieae*, which has been isolated in the flesh samples in the present study, is the etiological agent of lactococcosis, an ichthyopathology that affects rainbow trout farming. Thus, it would be important to prevent and control the presence of *L. garvieae* in fish farming [69,88]. 

In the viscera of rainbow trout, the presence and dominance of enterobacteria, primarily from the *Citrobacter* genus followed by the *Lactococcus* genus, aligns with the findings of Araújo et. al. [23], in which *Citrobacter* and *Lactococcus* became predominant in the intestine.

There was a notable distinction between the genera isolated from flesh and viscera samples, which is significant in terms of food quality and safety. While various species of *Lactococcus* (*L. lactis* and *L. cremoris*) were isolated from viscera, comprising 47.06% of the isolates, *Staphylococcus* (*S. warneri* and *S. hominis*) were predominantly found in flesh, accounting for up to 25% of the isolates. *S. warneri* (25%) was primarily identified in the rainbow trout fillet samples. This species can be found as part of the epithelial microbiota of rainbow trout, and it is not a direct pathogen. It is a commensal microorganism with the potential to become indirect pathobionts, promoting the growth and colonisation of the host by other pathogens when changes in the skin microbiota occur, potentially leading to the occurrence of diseases [89].

### 3.4. Distribution of Bacterial Genus among Type of Samples

**Figure 8:**
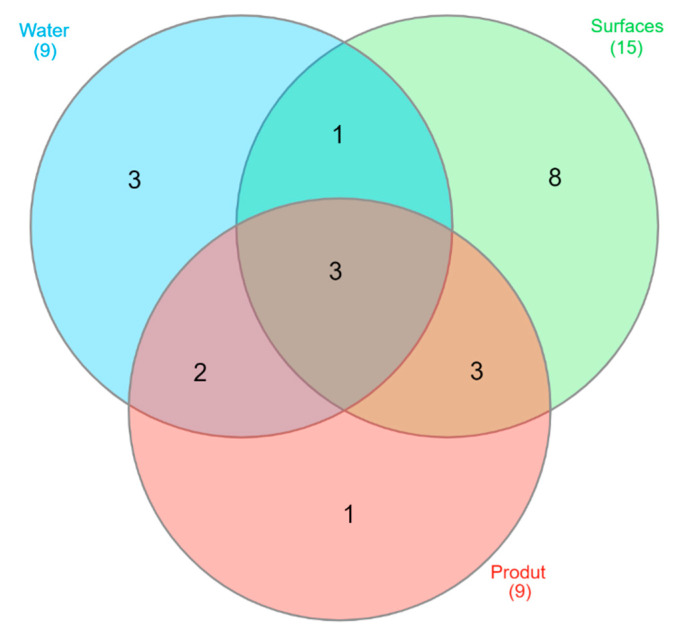
Venn diagram showing the relationship between the common bacterial groups in water, surfaces, and product samples.

Venn diagram (Figure 8) illustrates that, although 57% of the identified genera are unique to each type of sample, there is a substantial percentage of bacterial genera (43%) present in both environmental and rainbow trout samples. As indicated in Table 5, it can be seen that *Citrobacter*, *Hafnia*, and *Pseudomonas* were isolated from all types of samples (water, food-contact surfaces, or rainbow trout samples). However, the results of the molecular identification of the selected isolates (98 isolates) from water, food contact surfaces, and rainbow trout samples (flesh and viscera) are reflected in Table S7 (Supplementary Material) in the function of visits to the industry. Therefore, it is crucial to maintain good hygienic conditions throughout the rainbow trout processing, as well as implement good manufacturing practices to preserve the health status of farmed fish. 

## 4. Conclusions

This manuscript offers a comprehensive examination of the microbial status and potential contamination routes within the rainbow trout processing chain on the farm. The findings suggest that microbial transmission to the final product could have originated from various sources, including contaminated outlet water, as well as areas associated with slaughtering, gutting, and evisceration. While *L. monocytogenes* and *Salmonella* spp. were not detected in the analysed samples, other species such as *Aeromonas* spp., *Staphylococcus* spp., or *Pseudomonas* spp. were found in samples of contaminated water, food-contact surfaces, and trout.

In summary, the results indicate that the overall hygienic and sanitary conditions at the fish farm were deemed satisfactory. However, they underline the critical importance of monitoring both the physico-chemical and microbiological quality of rainbow trout production water, as well as the hygienic-sanitary conditions within the processing plant. These results serve as valuable clues for risk managers, highlighting the necessity of implementing targeted measures to safeguard the quality and safety of rainbow trout production, and ultimately ensuring consumer health and satisfaction.

## Figures and Tables

**Table 1:** Description of the analytical methods used for microbiological analyses of the samples from the fish farm.

Microorganism	Media/Enrichment	Media Supplement	ISO Standard	Analysed Samples	Incubation	
					T (°C)	Time (h)
Aerobic Mesophilic Bacteria	Plate Count Agar	-	4833-1 [37]	W, P, S, E	30	48
Coagulase-positive *Staphylococcus* (CPS)	Baird-Parker Agar (BP)	Egg Yolk Tellurite Emulsion	6888-1 [38]	P, S	37	24–48
Lactic Acid Bacteria (LAB)	De Man, Rogosa, Sharpe Agar (MRS)	-	15214 [39]	W, P	30 *	72
Yeast and molds	Rose-Bengal Chloramphenicol Agar (RBCA)	Chloramphenicol Supplement	21527-1 [40]	E, P	30	120
Total coliforms	Violet Red Bile Lactose Agar (VRBL)	-	4832 [41]	W, S	30	24
*Enterobacteriaceae*	Violet Red Bile Glucose Agar, (VRBG)	-	21528-2 [42]	W, P, S	37	24
Sulphite-reducing *Clostridium*	Perfringens Agar Base (TSC & SFP)	Egg Yolk Emulsion and Perfringens (TSC) selective supplement	15213-1 [43]	W, P	40 *	48
*Aeromonadaceae*	Aeromonas medium base (RYAN)	Ampicillin supplement	-	P	30–35	24
Psychrotrophic bacteria	Plate Count Agar	-	17410 [44]	P	7	24
*Enterococcaceae*	MacConkey agar No. 2	-	-	P	37	24
*Salmonella*	Xylose-Lysine-Deoxycholate (XLD) Agar	-	6579 [45]	P	37	24
	Rappaport-Vassiliadis Enrichment Broth		6579 [45]		41.5	24
*Listeria monocytogenes*	Chromogenic Listeria Agar, Ottaviani and Agosti (ALOA)	OCLA Selective Supplement/OCLA Differential	11290-1/2 [46]	W, P, S	37	24–48
	Listeria Selective Agar, Oxford formulation	Listeria selective Supplement	11290-1/2 [46]		37	24–48
	Fraser broth/Half-Fraser broth (FF/HF)	Half Fraser Supplement, Fraser Supplement	11290-1/2 [46]		37	24–48

All media selected were supplied by OXOID. W = Water samples; E = Environmental air samples; P: Product samples (flesh and viscera of rainbow trout) and S: Surfaces samples. * Incubation under anaerobic conditions of 10% CO_2_.

**Table 2:** Results of the physicochemical analysis (mean ± standard deviation obtained from the seven independent visits) of water and ice samples.

Samples	pH	Conductivity (µS/cm)	Salinity (ppm)	Total Dissolved Solids (TDS) (ppm)	Free Chlorine (ppm)
Input water	6.87 ± 0.81	783.40 ± 110.60	377.40 ± 53.24	530.60 ± 80.96	0.06 ± 0.04
Processing water	7.43 ± 0.25	870.50 ± 227.94	429.75 ± 120.08	583.00 ± 153. 22	0.35 ± 0.26
Output water	6.75 ± 0.54	853.00 ± 170.73	415.20 ± 85.59	570.80 ± 113.60	0.08 ± 0.12
Ice	7.05 ± 0.01	91.00 ± 0.01	50.00 ± 0.01	69.00 ± 0.01	ND *

* ND: Not detected.

**Table 3:** Significant correlations between the different FCS analysed (*p* < 0.05).

Microorganism	Surface 1	Surface 2	*r*	*p*
Aerobic Mesophilic Bacteria (AMB)	Filleting machine	Packaging box	0.87	0.01
	Evisceration machine	Packaging belt	0.85	0.01
	Packaging box	Packaging belt	0.76	0.05
Coliforms	Knife	Packaging box	0.99	0.00
	Washing belt	Filleting machine	−0.95	0.00
	Packaging belt	Packaging box	0.92	0.00
	Knife	Packaging belt	0.90	0.01
*Enterobacteriaceae*	Knife	Packaging belt	0.95	0.00
	Knife	Filleting machine	0.76	0.05
Coagulase-positive *Staphylococcus*	Slaughtering bath	Worker hands	0.92	0.00
	Evisceration machine	Packaging belt	0.89	0.01
	Slaughtering bath	Filleting machine	0.81	0.03
	Washing belt	Evisceration machine	−0.81	0.03

**Table 4:** Mean, standard deviation (S.D.), and 95% confidence intervals (log CFU/g) for aerobic mesophilic bacteria, psychrotrophic bacteria, molds and yeasts, lactic acid bacteria, *Enterococcus*, coagulase-positive *Staphylococcus*, *Clostridium*, *Enterobacteriaceae* counts in product samples (flesh and viscera).

Sample	Microorganism	Mean	S.D.	95% C.I.
Flesh	Aerobic mesophilic bacteria	3.22	1.67	[2.52–3.92]
	Psychrotrophic bacteria	4.28	1.71	[3.56–5.00]
	Molds and yeasts	0.94	1.57	[0.28–1.60]
	Lactic Acid Bacteria	2.70	1.77	[1.94–3.46]
	*Enterococcus*	2.10	1.85	[1.32–2.88]
	Coagulase-positive *Staphylococcus*	0.21	0.66	[−0.28–0.48]
	*Clostridium*	0.03	0.15	[−0.08–0.09]
	*Enterobacteriaceae*	0.62	1.28	[0.07–1.17]
	*Aeromonadaceae*	4.57	0.52	[3.82–5.31]
Viscera	Aerobic mesophilic bacteria	3.97	1.77	[3.21–4.73]
	Psychrotrophic bacteria	4.43	1.40	[3.83–5.03]
	Molds and yeasts	1.04	1.40	[0.46–1.63]
	Lactic Acid Bacteria	2.98	1.43	[2.38–3.58]
	*Enterococcus* spp.	2.61	2.00	[1.75–3.47]
	Coagulase-positive *Staphylococcus*	0.08	0.37	[−0.19–0.24]
	*Clostridium*	0.51	1.12	[0.04–0.98]
	*Enterobacteriaceae*	1.39	1.73	[0.65–2.13]
	*Aeromonadaceae*	5.16	0.35	[4.75–5.56]

**Table 5:** Summary of bacterial genera shared between water samples, food contact surfaces, and rainbow trout.

Sample	Genus
Water	*Escherichia*, *Serratia*, *Enterococcus*
Surfaces	*Macrococcus*, *Brevibacterium*, *Shewanella*, *Erwinia*, *Klebsiella*, *Lysinibacillus*, *Morganella*, *Pantoea*
Product	*Aeromonas*
Water and Product	*Enterobacter*, *Lactococcus*
Surfaces and Product	*Staphylococcus*, *Providencia*, *Kluyvera*
Water and Surfaces and Product	*Citrobacter*, *Hafnia*, *Pseudomonas*
Water and Surfaces	*Exiguobacterium*

## Data Availability

The data used to support the findings of this study can be made available by the corresponding author upon request.

## Supplementary Materials

The following supporting information can be downloaded at: https://www.mdpi.com/article/10.3390/foods12203718/s1, Table S1: Mean, standard deviation (S.D.) and 95% confidence intervals (log CFU/mL) for aerobic mesophilic bacteria (AMB), *Enterobacteriaceae*, lactic acid bacteria (LAB), total coliforms and sulfite-reducing *Clostridium* (SRC) in the water samples analysed in the different processing areas; Table S2: Mean, standard deviation (S.D.) and 95% confidence intervals (log CFU/mL) for the aerobic mesophilic bacteria, *Enterobacteriaceae*, lactic acid bacteria, total coliforms, and sulphite-reducing *Clostridium* counts in water according to the seasonal variations; Table S3: Mean, standard deviation (S.D.) and 95% confidence intervals (CFU/m^3^) for the aerobic mesophilic bacteria and molds and yeasts counts in environmental air samples in the analysed processing areas; Table S4: Mean, standard deviation (S.D.) and 95% confidence intervals (CFU/m^3^) for the aerobic mesophilic bacteria and molds and yeasts counts in environmental air samples according to the seasonal variations; Table S5: Mean, standard deviation (S.D.) and 95% confidence intervals (log CFU/cm^2^) for the aerobic mesophilic bacteria, coliforms, *Enterobacteriaceae*, coagulase-positive *Staphylococcus* counts in food-contact surfaces samples in the analysed processing areas; Table S6: Mean, standard deviation (S.D.) and (95%) confidence intervals (log CFU/cm^2^) for the aerobic mesophilic bacteria, coliforms, *Enterobacteriaceae*, coagulase-positive *Staphylococcus* counts in FCS samples in the analysed processing areas according to the seasons of the year. Table S7: Molecular identification (16S rRNA gene PCR) of the 99 isolates selected based on visits to the industry (I–VII), category (water, food contact surfaces, and rainbow trout), and type of sample of each category.
