# Supplementary material for: Tracking Microbial Diversity and Hygienic-Sanitary Status during Processing of Farmed Rainbow Trout (Oncorhynchus mykiss)
Source: Foods. 2023 Oct 10;12(20):3718. doi: 10.3390/foods12203718 (PMC10606590; doi:10.3390/foods12203718)
Supplement: Supplementary file 1 [file foods-12-03718-s001.zip › foods-2639356-supplementary.pdf]

foods

MDPI

Table S1. Mean, standard deviation (S.D.) and 95 % confidence intervals (log CFU/mL) for aerobic mesophilic bacteria (AMB), *Enterobacteriaceae*, lactic acid bacteria (LAB), total coliforms and sulfite-reducing *Clostridium* spp (SRC) in the water samples analyzed in the different processing areas.

Sample	Microorganism	Mean	S.D.	95 % C.I.
Input water	Aerobic Mesophilic bacteria	2.96	0.68	[2.45 - 3.47]
	Enterobacteriaceae	1.12	0.89	[0.46 - 1.78]
	Lactic acid bacteria	0.31	0.42	[0 - 0.62]
	Coliforms	1.38	0.46	[1.03 - 1.73]
	Sulfite-reducing *Clostridium* spp.	0.41	0.36	[0.14 - 0.68]
Output water	Aerobic Mesophilic bacteria	4.23	0.62	[3.76 - 4.70]
	Enterobacteriaceae	2.56	1.24	[1.64 - 3.48]
	Lactic acid bacteria	1.82	1.47	[0.73 - 2.91]
	Coliforms	2.83	0.52	[2.44 - 3.22]
	Sulfite-reducing *Clostridium* spp.	0.23	0.61	[-0.22 - 0.68]
Processing water	Aerobic Mesophilic bacteria	0.31	0.58	[-0.12 - 0.74]
	Enterobacteriaceae	0.24	0.64	[-0.23 - 0.71]
	Lactic acid bacteria	0.10	0.26	[-0.10 - 0.30]
	Coliforms	ND *	ND	ND
	Sulfite-reducing *Clostridium* spp.	ND	ND	ND

* ND: Not detected in the analyzed samples (< 1 CFU/mL).

Table S2. Mean, standard deviation (S.D.) and 95 % confidence intervals (log CFU/mL) for the aerobic mesophilic bacteria, *Enterobacteriaceae*, lactic acid bacteria, total coliforms, and sulphite-reducing *Clostridium* spp. counts in water according to the seasonal variations.

Season	Microorganism	Mean	S.D.	C.I. (95 %)
Autumn	Aerobic Mesophilic bacteria	2.82	2.32	[0.97 - 4.67]
	Enterobacteriaceae	0.94	1.60	[-0.25 - 2.21]
	Lactic Acid Bacteria	0.58	0.75	[-0.03 - 1.18]
	Total coliforms	1.57	1.52	[0.36 - 2.78]
	Sulfite-reducing *Clostridium* spp.	ND *	ND	ND
Winter	Aerobic Mesophilic bacteria	2.36	1.78	[0.94 - 3.78]
	Enterobacteriaceae	1.43	1.19	[0.47 - 2.39]
	Lactic Acid Bacteria	0.57	1.39	[-0.54 -1.68]
	Total coliforms	1.27	1.27	[0.26 - 2.28]
	Sulfite-reducing *Clostridium* spp.	0.15	0.25	[-0.03 - 0.35]
Spring	Aerobic Mesophilic bacteria	2.38	1.46	[1.21 - 3.55]
	Enterobacteriaceae	1.29	1.32	[0.24 - 2.34]
	Lactic Acid Bacteria	1.45	1.36	[0.36 - 2.54]
	Total coliforms	1.26	1.13	[0.36 - 2.16]
	Sulfite-reducing *Clostridium* spp.	0.25	0.42	[-0.06 - 0.58]
Summer	Aerobic Mesophilic bacteria	2.36	2.07	[0.02 - 4.70]
	Enterobacteriaceae	1.84	1.61	[0.03 - 3.65]
	Lactic Acid Bacteria	ND	ND	ND
	Total coliforms	1.64	1.49	[0.54 - 3.32]
	Sulfite-reducing *Clostridium* spp.	0.69	0.82	[0.08 - 1.61]

* ND: Not detected in the analyzed samples (<1 CFU/mL).

Table S3. Mean, standard deviation (S.D.) and 95 % confidence intervals (CFU/m^3) for the aerobic mesophilic bacteria and molds and yeasts counts in environmental air samples in the analyzed processing areas.

Sampling area	Microorganism	Mean	S.D.	95 % C. I.
Slaughtering area	Aerobic Mesophilic bacteria	2.09	1.01	[1.35 - 2.83]
	Molds and yeasts	1.18	0.69	[0.67 - 1.68]
Gutting area	Aerobic Mesophilic bacteria	2.41	1.10	[1.59 - 3.22]
	Molds and yeasts	1.20	0.93	[0.51 - 1.88]
Packaging area	Aerobic Mesophilic bacteria	2.08	0.97	[1.35 - 2.80]
	Molds and yeasts	0.94	0.83	[0.33 - 1.54]

Table S4. Mean, standard deviation (S.D.) and 95 % confidence intervals (CFU/m^3) for the aerobic mesophilic bacteria and molds and yeasts counts in environmental air samples according to the seasonal variations.

Season	Microorganism	Mean	S.D.	95 % C.I.
Autumn	Aerobic mesophilic bacteria	2.14	0.29	[1.90-2.37]
	Molds and yeasts	1.22	0.31	[0.97-1.47]
Winter	Aerobic mesophilic bacteria	1.31	1.43	[0.16-2.46]
	Molds and yeasts	0.43	0.50	[0.02-0.84]
Spring	Aerobic mesophilic bacteria	2.83	0.33	[2.58-3.08]
	Molds and yeasts	1.19	1.00	[0.39-1.99]
Summer	Aerobic mesophilic bacteria	2.79	0.25	[2.52-3.06]
	Molds and yeasts	2.07	0.11	[1.95-2.19]

Table S5. Mean, standard deviation (S.D.) and 95 % confidence intervals (log CFU/cm^2) for the aerobic mesophilic bacteria, coliforms, *Enterobacteriaceae*, coagulase-positive *Staphylococcus* spp. counts in food-contact surfaces samples in the analyzed processing areas.

Sampling area	Microorganism	Mean	S.D.	95 % C.I.
Slaughtering bath	Aerobic mesophilic bacteria	1.96	1.49	[1.30 - 2.62]
	Coliforms	1.01	0.57	[0.23 - 1.79]
	Enterobacteriaceae	0.60	1.27	[0.44 - 0.76]
	Coagulase-positive *Staphylococcus* spp.	0.06	1.27	[-0.88- 0.61]
Washing belt	Aerobic mesophilic bacteria	1.2	0.96	[0.50 -1.90]
	Coliforms	-0.35 *	0.91	[-1.02 - 0.31]
	Enterobacteriaceae	-0.29 *	0.42	[-0.60 - 0.02]
	Coagulase-positive *Staphylococcus* spp.	0.07	0.2	[-0.07 - 0.21]
Gutting machine	Aerobic mesophilic bacteria	1.83	1.45	[0.76 - 2.90]
	Coliforms	0.71	0.99	[-0.02 - 1.43]
	Enterobacteriaceae	0.84	0.94	[0.14 - 1.54]
	Coagulase-positive *Staphylococcus* spp.	0.24	0.96	[-0.47 - 0.94]
Knife	Aerobic mesophilic bacteria	0.76	1.51	[-0.35 - 1.87]
	Coliforms	0.30	0.70	[-0.21 - 0.81]
	Enterobacteriaceae	0.08	0.21	[-0.08 - 0.24]

	Coagulase-positive *Staphylococcus* spp.	ND *	ND	ND
Worker hands	Aerobic mesophilic bacteria	1.23	0.92	[0.55 - 1.91]
	Coliforms	0.12	0.3	[-0.10 - 0.35]
	Enterobacteriaceae	0.13	0.34	[-0.12 - 0.38]
	Coagulase-positive *Staphylococcus* spp.	0.25	1.13	[0.59 - 1.09]
Packaging belt	Aerobic mesophilic bacteria	0.86	0.89	[0.20 - 1.52]
	Coliforms	0.74	1.06	[-0.05 - 1.52]
	Enterobacteriaceae	0.08	0.21	[-0.08 - 0.24]
	Coagulase-positive *Staphylococcus* spp.	0.37	0.74	[-0.18 - 0.92]
Filleting machine	Aerobic mesophilic bacteria	0.91	0.95	[0.21 - 1.61]
	Coliforms	0.23	0.73	[-0.31 - 0.78]
	Enterobacteriaceae	0.57	1.21	[-0.33 - 1.47]
	Coagulase-positive *Staphylococcus* spp.	0.09	0.24	[-0.09 - 0.27]
Packaging box	Aerobic mesophilic bacteria	1.08	1.39	[0.07 - 2.09]
	Coliforms	0.04	0.11	[-0.04 - 0.12]
	Enterobacteriaceae	ND	ND	ND
	Coagulase-positive *Staphylococcus* spp.	ND	ND	ND
Packaged fillet conveyor belt	Aerobic mesophilic bacteria	0.86	0.89	[0.20 - 1.52]
	Coliforms	0.74	0.74	[0.19 - 1.52]
	Enterobacteriaceae	0.08	0.08	[0.02 - 0.24]
	Coagulase-positive *Staphylococcus* spp.	0.37	0.37	[0.10 - 0.92]

ND *: Not detected (< 1 CFU/cm^2).

* The results expressed as " * " were below the detection limit of the technique applied for enumeration in each case.

Table S6. Mean, standard deviation (S.D.) and (95 %) confidence intervals (log CFU/ cm^2) for the aerobic mesophilic bacteria, coliforms, *Enterobacteriaceae*, coagulase-positive *Staphylococcus* spp. counts in FCS samples in the analyzed processing areas according to the seasons of the year.

Season	Microorganism	Mean	S.D.	95 % C.I.
Autumn	Mesophilic bacteria	2.04	1.42	[1.40 - 2.68]
	Coliforms	0.59	0.87	[0.20 - 0.98]
	Enterobacteriaceae	0.39	0.75	[0.04 - 0.74]
	Coagulase-positive *Staphylococcus* spp.	0.24	0.59	[-0.03 - 0.51]
Winter	Mesophilic bacteria	0.89	1.2	[0.34 - 1.44]
	Coliforms	0.30	0.64	[0.01 -0.59]
	Enterobacteriaceae	0.25	0.66	[-0.06 - 0.56]
	Coagulase-positive *Staphylococcus* spp.	0.07	0.28	[-0.07 - 0.21]
Spring	Mesophilic bacteria	0.78	1.23	[0.21 - 1.35]
	Coliforms	0.16	0.8	[-0.21 - 0.53]
	Enterobacteriaceae	0.03	0.82	[-0.34 - 0.40]
	Coagulase-positive *Staphylococcus* spp.	-0.12	1.11	[-0.63 - 0.39]
Summer	Mesophilic bacteria	1.16	0.86	[0.59 - 1.73]
	Coliforms	0.1	1.14	[-0.64 - 0.84]
	Enterobacteriaceae	0.35	0.71	[-0.12 - 0.82]
	Coagulase-positive *Staphylococcus* spp.	0.68	0.96	[0.06 - 1.30]

Table S7. Molecular identification (16S rRNA gene PCR) of the 98 isolates selected based on visits to the industry (I – VII), category (water, food contact surfaces and rainbow trout) and type of sample of each category.

Visit No.	Category	Sample type	Isolated ID	[a] Identified species/genus	Family	Order	Query Leng (bp)	Query ID	Max Score	Total Score	Per. Query Cover (%)	E value	Per. Ident (%)	Accession Number (NCBI GenBank)
I	Water	Input water	1	*Citrobacter gillenii*	*Enterobacteriaceae*	*Enterobacterales*	739	60321	1365	1365	100	0.0	100,00	gi|1751390857|MN513228.1
I	Water	Input water	12	*Hafnia paralvei*	*Hafniaceae*	*Enterobacterales*	819	452003	1513	11958	100	0.0	100,00	gi|1344462186|CP014031.2
I	Water	Output water	5	*Citrobacter gillenii*	*Enterobacteriaceae*	*Enterobacterales*	749	378843	1378	1378	100	0.0	99,87	gi|1751390857|MN513228.1
I	Product	Flesh	20	*Citrobacter freundii*	*Enterobacteriaceae*	*Enterobacterales*	659	24609	1218	9744	100	0.0	100,00	gi|1615107120|CP038856.1
I	Product	Viscera	18	*Citrobacter freundii*	*Enterobacteriaceae*	*Enterobacterales*	679	222855	1254	1254	100	0.0	100,00	gi|1767042976|MN582981.1
I	Surfaces	Gutting machine	15	*Hafnia paralvei*	*Hafniaceae*	*Enterobacterales*	729	495443	1341	1341	100	0.0	99,86	gi|1207851012|KY849249.1
I	Surfaces	Gutting machine	22	*Hafnia paralvei*	*Hafniaceae*	*Enterobacterales*	649	42343	1199	1199	100	0.0	100,00	gi|1370745659|MH119676.1
I	Surfaces	Packaged fillets belt	21	*Providencia rustigianii*	*Morganellaceae*	*Enterobacterales*	649	15431	1199	8325	100	0.0	100,00	gi|1539485721|LR134189.1
II	Product	Flesh	17	*Staphylococcus warneri*	*Staphylococcaceae*	*Bacillales*	699	24661	1291	1291	100	0.0	100,00	gi|1859633578|MT642942.1
II	Product	Viscera	16	*Enterobacter sp.*	*Enterobacteriaceae*	*Enterobacterales*	719	40339	1328	1328	100	0.0	100,00	gi|1434133245|MH669333.1
II	Surfaces	Worker hands	6	*Staphylococcus warneri*	*Staphylococcaceae*	*Bacillales*	769	61399	1421	1421	100	0.0	100,00	gi|1859633578|MT642942.1
III	Surfaces	Packaging belt	8	*Staphylococcus aureus*	*Staphylococcaceae*	*Bacillales*	819	62179	1513	9027	100	0.0	100,00	gi|2032998575|CP071943.1
III	Surfaces	Packaging belt	9	*Macrococcus caseolyticus*	*Staphylococcaceae*	*Bacillales*	869	112043	1605	1605	100	0.0	100,00	gi|1822211978|MT225659.1
III	Surfaces	Packaged fillets belt	11	*Staphylococcus aureus*	*Staphylococcaceae*	*Bacillales*	869	46643	1605	1605	100	0.0	100,00	gi|2032998575|CP071943.1
IV	Water	Input water	27	*Serratia marcescens*	*Yersiniaceae*	*Enterobacterales*	949	24137	1753	12225	100	0.0	100,00	gi|1860418725|CP055161.1
IV	Water	Output water	28	*Enterococcus sp*	*Enterococcaceae*	*Lactobacillales*	1179	45869	2178	2178	100	0.0	100,00	gi|2103567986|OK326653.1
IV	Product	Flesh	9	*Citrobacter gillenii*	*Enterobacteriaceae*	*Enterobacterales*	937	63393	1725	1725	100	0.0	99,89	gi|1311515739|MG675590.1
IV	Product	Flesh	14	*Staphylococcus sp*	*Staphylococcaceae*	*Bacillales*	951	61843	1746	1746	100	0.0	99,79	gi|1359912432|MH057213.1
IV	Product	Flesh	15	*Staphylococcus warneri*	*Staphylococcaceae*	*Bacillales*	1090	36033	2013	2013	100	0.0	100,00	gi|1840136874|MT448999.1
IV	Product	Flesh	16	*Staphylococcus warneri*	*Staphylococcaceae*	*Bacillales*	971	6373	1794	1794	100	0.0	100,00	gi|1859633578|MT642942.1
IV	Product	Flesh	17	*Staphylococcus hominis*	*Staphylococcaceae*	*Bacillales*	973	61815	1797	1797	100	0.0	100,00	gi|1851774560|MT585539.1
IV	Product	Viscera	1	*Lactococcus lactis*	*Streptococcaceae*	*Lactobacillales*	901	59401	1664	1664	100	0.0	100,00	gi|1743123904|MN466963.1
IV	Product	Viscera	2	*Lactococcus lactis*	*Streptococcaceae*	*Lactobacillales*	1088	4879	1989	1989	100	0.0	99,91	gi|1743123904|MN466963.1
IV	Product	Viscera	3	*Lactococcus lactis*	*Streptococcaceae*	*Lactobacillales*	354	19409	654	654	100	0.0	100,00	gi|1843990348|MT498452.1

IV	Product	Viscera	4	*Staphylococcus epidermis*	*Staphylococcaceae*	*Bacillales*	970	27371	1792	1792	100	0.0	100,00	gi|2105350976|OK355385.1
IV	Product	Viscera	5	*Lactococcus lactis*	*Streptococcaceae*	*Lactobacillales*	1078	535463	2059	2059	100	0.0	99,91	gi|1852325286|MT597705.1
IV	Product	Viscera	7	*Lactococcus cremoris*	*Streptococcaceae*	*Lactobacillales*	1129	51855	2067	12405	100	0.0	99,65	gi|1834244649|CP051518.1
IV	Product	Viscera	8	*Lactococcus cremoris*	*Streptococcaceae*	*Lactobacillales*	1248	58791	2213	13280	99	0.0	98,57	gi|1834244649|CP051518.1
IV	Product	Viscera	8b	*Lactococcus cremoris*	*Streptococcaceae*	*Lactobacillales*	1182	34945	2145	12870	99	0.0	99,33	gi|1834244649|CP051518.1
IV	Product	Viscera	11	*Citrobacter gillenii*	*Enterobacteriaceae*	*Enterobacterales*	978	5265	1807	1807	100	0.0	100,00	gi|1707325058|MN197983.1
IV	Product	Viscera	12	*Citrobacter freundii*	*Enterobacteriaceae*	*Enterobacterales*	1080	59783	1995	15964	100	0.0	100,00	gi|1881832576|CP057509.
IV	Product	Viscera	13	*Citrobacter sp*	*Enterobacteriaceae*	*Enterobacterales*	980	49097	1790	1790	99	0.0	99,69	gi|1707325058|MN197983.1
IV	Surfaces	Gutting machine	18	*Providencia rustigianii*	*Morganellaceae*	*Enterobacterales*	889	1519	1637	1637	100	0.0	99,89	gi|2077896939|MZ734440.1
IV	Surfaces	Gutting machine	24	*Shewanella sp*	*Shewanellaceae*	*Alteromonadales*	1028	39665	1899	16810	100	0.0	100,00	gi|1356669658|CP022089.2
IV	Surfaces	Knife	19	*Morganella morganii*	*Enterobacteriaceae*	*Enterobacterales*	1009	31517	1858	1858	100	0.0	99,90	gi|1779076118|MN744697.1
IV	Surfaces	Slaughter	20	*Citrobacter sp*	*Enterobacteriaceae*	*Enterobacterales*	1345	19785	2172	17382	93	0.0	97,50	gi|1615107120|CP038856.1
IV	Surfaces	Packaged fillets belt	21	*Kluyvera sp*	*Enterobacteriaceae*	*Enterobacterales*	939	49157	1712	1712	100	0.0	99,57	gi|1826706563|MT263028.1
IV	Surfaces	Packaged fillets belt	25	*Macrococcus caseolyticus*	*Staphylococcaceae*	*Bacillales*	1076	53417	1965	1965	99	0.0	99,63	gi|1682060885|MN062068.1
IV	Surfaces	Clean box	22	*Klebsiella sp*	*Enterobacteriaceae*	*Enterobacterales*	1360	49735	2163	2163	91	0.0	97,70	gi|1782828319|MN812670.1
IV	Surfaces	Packaging belt	23	*Citrobacter freundii*	*Enterobacteriaceae*	*Enterobacterales*	1008	6417	1851	14811	100	0.0	99,80	gi|1615107120|CP038856.1
IV	Surfaces	Packaging belt	26	*Macrococcus caseolyticus*	*Staphylococcaceae*	*Bacillales*	978	45319	1794	1794	100	0.0	99,80	gi|1725274959|MK443060.1
VI	Water	Input water	4	*Enterobacter ludwigii*	*Enterobacteriaceae*	*Enterobacterales*	1004	619	1855	1855	100	0	100,00	gi|688474302|KJ850208.1
VI	Water	Input water	5	*Escherichia coli*	*Enterobacteriaceae*	*Enterobacterales*	1079	378623	1980	13831	100	0	99,81	gi|1836698516|CP052057.1
VI	Water	Input water	18	*Citrobacter gillenii*	*Enterobacteriaceae*	*Enterobacterales*	999	26655	1845	1845	100	0	100,00	gi|1751390857|MN513228.1
VI	Water	Input water	19	*Enterobacter ludwigii*	*Enterobacteriaceae*	*Enterobacterales*	1026	35323	1888	15012	99	0	99,90	gi|1822110488|CP039741.1
VI	Water	Output water	6	*Hafnia alvei*	*Hafniaceae*	*Enterobacterales*	973	50197	1797	1797	100	0.0	100,00	gi|1830434209|MT323144.1
VI	Water	Output water	7	*Hafnia paralvei*	*Hafniaceae*	*Enterobacterales*	896	34483	1644	13062	100	0	99,89	gi|1344462186|CP014031.2
VI	Water	Output water	8	*Citrobacter gillenii*	*Enterobacteriaceae*	*Enterobacterales*	1008	48747	1862	1862	100	0	100,00	gi|2088097036|MZ964583.1
VI	Water	Output water	9	*Hafnia paralvei*	*Hafniaceae*	*Enterobacterales*	1015	21105	1873	1873	99	0	100,00	gi|1517398458|MG996519.1
VI	Water	Output water	14	*Enterobacter ludwigii*	*Enterobacteriaceae*	*Enterobacterales*	969	52883	1784	14185	100	0	99,90	gi|1822110488|CP039741.1
VI	Product	Flesh	RB	*Pseudomonas laurentiana*	*Pseudomonadaceae*	*Pseudomonadales*	739	15689	1365	1365	100	0.0	100,00	gi|1858593063|MT626819.1
VI	Product	Flesh	RC	*Pseudomonas gessardii*	*Pseudomonadaceae*	*Pseudomonadales*	951	36699	1753	1753	99	0.0	100,00	gi|1858593069|MT626825.1

VI	Product	Viscera	RD	*Aeromonas media*	*Aeromonadaceae*	*Aeromonadales*	882	5405	1629	16276	100	0.0	100,00	gi|1841681316|CP038441.1
VI	Product	Viscera	McE	*Aeromonas sp.*	*Aeromonadaceae*	*Aeromonadales*	933	65095	1714	1714	100	0.0	99,79	gi|1835439075|MT396438.1
VI	Surfaces	Wash tape	1	*Hafnia paralvei*	*Hafniaceae*	*Enterobacterales*	878	10123	1622	12874	100	0	100,00	gi|1344462186|CP014031.2
VI	Surfaces	Wash tape	10	*Exiguobacterium sp.*	*Bacillaceae*	*Bacillales*	974	53313	1796	1796	99	0	100,00	gi|1041495566|KU758895.1
VI	Surfaces	Wash tape	12	*Staphylococcus aureus*	*Staphylococcaceae*	*Bacillales*	974	60795	1799	1799	100	0	100,00	gi|2222591213|ON222796.1
VI	Surfaces	Gutting machine	2	*Hafnia paralvei*	*Hafniaceae*	*Enterobacterales*	995	19251	1838	14558	100	0	100,00	gi|1344462186|CP014031.2
VI	Surfaces	Gutting machine	15	*Pseudomonas psychrophila*	*Pseudomonadaceae*	*Pseudomonadales*	1027	39523	1892	1892	100	0	99,90	gi|1772803573|LC508010.1
VI	Surfaces	Gutting machine	16	*Pseudomonas psychrophila*	*Pseudomonadaceae*	*Pseudomonadales*	1136	20427	2093	2093	100	0	99,91	gi|1772803573|LC508010.1
VI	Surfaces	Slaughter	3	*Hafnia paralvei*	*Hafniaceae*	*Enterobacterales*	1010	17213	1866	14824	100	0	100,00	gi|1344462186|CP014031.2
VI	Surfaces	Filleting machine	17	*Citrobacter gillenii*	*Enterobacteriaceae*	*Enterobacterales*	959	17365	1772	1772	100	0	100,00	gi|1311515739|MG675590.1
VII	Water	Input water	5	*Pseudomonas aeruginosa*	*Pseudomonadaceae*	*Pseudomonadales*	945	41647	1746	1746	100	0.0	100,00	gi|1722681544|MN309895.1
VII	Water	Input water	7	*Escherichia fergusonii*	*Enterobacteriaceae*	*Enterobacterales*	956	63823	1718	1718	97	0.0	99,89	gi|1836146575|MT416422.1
VII	Water	Input water	8	*Serratia marcescens*	*Yersiniaceae*	*Enterobacterales*	865	22757	1592	11100	100	0.0	99,88	gi|1860418725|CP055161.1
VII	Water	Input water	13	*Escherichia coli*	*Enterobacteriaceae*	*Enterobacterales*	906	12611	1670	11665	99	0.0	100,00	gi|2249798319|CP098214.1
VII	Water	Output water	1	*Lactococcus garvieae*	*Streptococcaceae*	*Lactobacillales*	922	29985	1698	1698	99	0.0	100,00	gi|1852867376|MT604772.1
VII	Water	Output water	2	*Enterococcus hirae*	*Enterococcaceae*	*Lactobacillales*	933	62895	1718	10311	100	0.0	99,89	gi|1861262708|CP055232.1
VII	Water	Output water	3	*Exiguobacterium undae*	*Bacillaceae*	*Bacillales*	946	20995	1748	1748	100	0.0	100,00	gi|1605306739|MK414802.1
VII	Water	Output water	4	*Pseudomonas aeruginosa*	*Pseudomonadaceae*	*Pseudomonadales*	957	56125	1768	1768	100	0.0	100,00	gi|1723195072|MN314670.1
VII	Water	Output water	9	*Escherichia coli*	*Enterobacteriaceae*	*Enterobacterales*	915	34261	1690	11780	100	0.0	100,00	gi|2244114683|OW968111.1
VII	Water	Output water	10	*Serratia marcescens*	*Yersiniaceae*	*Enterobacterales*	729	43779	1321	1321	100	0.0	99,31	gi|1908124013|MW031836.1
VII	Water	Output water	11	*Citrobacter arsenatis*	*Enterobacteriaceae*	*Enterobacterales*	678	19843	1242	1242	100	0.0	99,71	gi|1531243458|MK262983.1
VII	Water	Output water	12	*Hafnia paralvei*	*Hafniaceae*	*Enterobacterales*	931	28279	1720	13657	100	0.0	100,00	gi|1344462186|CP014031.2
VII	Product	Flesh	28	*Hafnia alvei*	*Hafniaceae*	*Enterobacterales*	1006	64181	1851	1851	99	0	99,90	gi|1535530918|MK294248.1
VII	Product	Flesh	29	*Providencia rettgeri*	*Morganellaceae*	*Enterobacterales*	1141	3681	2098	14625	99	0	99,91	gi|827747822|KR919605.1
VII	Product	Flesh	30	*Citrobacter freundii*	*Enterobacteriaceae*	*Enterobacterales*	960	29345	1766	1766	100	0	99,90	gi|731188935|KM507607.1
VII	Product	Flesh	31	*Hafnia paralvei*	*Hafniaceae*	*Enterobacterales*	1012	58529	1869	14809	100	0	100,00	gi|731188935|KM507607.1
VII	Product	Flesh	32	*Hafnia sp.*	*Hafniaceae*	*Enterobacterales*	1014	44831	1862	1862	100	0	99,80	gi|1850169240|MT573107.1
VII	Product	Flesh	33	*Hafnia sp.*	*Hafniaceae*	*Enterobacterales*	1004	36145	1855	1855	100	0	100,00	gi|1493570076|MK070120.1

VII	Product	Flesh	35	*Enterobacter sp.*	*Enterobacteriaceae*	*Enterobacterales*	958	201173	1764	1764	100	0	99,90	gi|1229082282|MF370900.1
VII	Product	Flesh	36	*Enterobacter cloacae*	*Enterobacteriaceae*	*Enterobacterales*	1014	15853	1873	1873	100	0	100,00	gi|1509786937|MH769021.1
VII	Product	Flesh	37	*Citrobacter gillenii*	*Enterobacteriaceae*	*Enterobacterales*	1000	65015	1847	1847	100	0	100,00	gi|1751390857|MN513228.1
VII	Product	Flesh	41	*Lactococcus garvieae*	*Streptococcaceae*	*Lactobacillales*	1041	51763	1923	1923	100	0	100,00	gi|1434131720|MH669171.1
VII	Product	Flesh	42	*Lactococcus garvieae*	*Streptococcaceae*	*Lactobacillales*	949	52139	1753	1753	100	0	100,00	gi|2058478868|CP077317.1
VII	Product	Viscera	34	*Kluyvera intermedia*	*Enterobacteriaceae*	*Enterobacterales*	885	55375	1628	13024	100	0	99,89	gi|1344462186|CP014031.2
VII	Product	Viscera	38	*Lactococcus cremoris*	*Streptococcaceae*	*Lactobacillales*	959	15051	1772	1772	100	0	100,00	gi|1344462186|CP014031.2
VII	Surfaces	Slaughter	15	*Shewanella seohaensis*	*Shewanellaceae*	*Alteromonadales*	920	30007	1700	1700	100	0.0	100,00	gi|1767042976|MN582981.1
VII	Surfaces	Slaughter	19	*Brevibacterium sp.*	*Brevibacteriaceae*	*Actinomycetales*	922	4299	1692	1692	100	0.0	99,78	gi|1434131730|MH669181.1
VII	Surfaces	Slaughter	21	*Erwinia sp.*	*Erwiniaceae*	*Enterobacterales*	914	23641	1687	1687	99	0.0	100,00	gi|1434172748|MH671652.1
VII	Surfaces	Slaughter	22	*Hafnia paralvei*	*Hafniaceae*	*Enterobacterales*	916	19647	1687	13336	100	0.0	99,89	gi|1835259294|MT386196.1
VII	Surfaces	Slaughter	24	*Pantoea sp.*	*Erwiniaceae*	*Enterobacterales*	624	54827	1118	1118	100	0.0	98,88	gi|1847518191|MT544710.1
VII	Surfaces	Worker hands	20	*Staphylococcus warneri*	*Staphylococcaceae*	*Bacillales*	902	53013	1661	1661	100	0.0	99,89	gi|1774529443|CP045843.1
VII	Surfaces	Filleting machine	16	*Lysinibacillus sphaericus*	*Bacillaceae*	*Bacillales*	918	10627	1687	1687	99	0.0	99,89	gi|1344462186|CP014031.2
VII	Surfaces	Filleting machine	18	*Brevibacterium sp.*	*Brevibacteriaceae*	*Actinomycetales*	923	62703	1683	1683	100	0.0	99,57	gi|1434131712|MH669163.1
VII	Surfaces	Filleting machine	23	*Providencia alcalifaciens*	*Morganellaceae*	*Enterobacterales*	923	7245	1703	1703	99	0.0	100,00	gi|1311515739|MG675590.1
VII	Surfaces	Filleting machine	26	*Citrobacter gillenii*	*Enterobacteriaceae*	*Enterobacterales*	903	6821	1668	1668	100	0.0	100,00	gi|1852867376|MT604772.1
VII	Surfaces	Filleting machine	27	*Hafnia paralvei*	*Hafniaceae*	*Enterobacterales*	917	45995	1688	13351	100	0.0	99,89	gi|1855411423|MT611574.1

[a] Closest related species found in the NCBI GenBank database.
